# Impaired Glucose Tolerance in Sleep Disorders

**DOI:** 10.1371/journal.pone.0009444

**Published:** 2010-03-01

**Authors:** Marietta Keckeis, Zuzana Lattova, Eszter Maurovich-Horvat, Pierre A. Beitinger, Steffen Birkmann, Christoph J. Lauer, Thomas C. Wetter, Johanna Wilde-Frenz, Thomas Pollmächer

**Affiliations:** 1 Max Planck Institute of Psychiatry, Munich, Germany; 2 Klinikum Ingolstadt, Centre of Mental Health, Ingolstadt, Germany; The Mental Health Research Institute of Victoria, Australia

## Abstract

**Background:**

Recent epidemiological and experimental data suggest a negative influence of shortened or disturbed night sleep on glucose tolerance. Due to the high prevalence of sleep disorders this might be a major health issue. However, no comparative studies of carbohydrate metabolism have been conducted in clinical sleep disorders.

**Methodology/Principal Findings:**

We performed oral glucose tolerance tests (OGTT) and assessed additional parameters of carbohydrate metabolism in patients suffering from obstructive sleep apnea syndrome (OSAS, N = 25), restless legs syndrome (RLS, N = 18) or primary insomnia (N = 21), and in healthy controls (N = 33). Compared to controls, increased rates of impaired glucose tolerance were found in OSAS (OR: 4.9) and RLS (OR: 4.7) patients, but not in primary insomnia patients (OR: 1.6). In addition, HbA1c values were significantly increased in the same two patient groups. Significant positive correlations were found between 2-h plasma glucose values measured during the OGTT and the apnea-arousal-index in OSAS (r = 0.56; p<0.05) and the periodic leg movement-arousal-index in RLS (r = 0.56, p<0.05), respectively. Sleep duration and other quantitative aspects of sleep were similar between patient groups.

**Conclusions/Significance:**

Our findings suggest that some, but not all sleep disorders considerably compromise glucose metabolism. Repeated arousals during sleep might be a pivotal causative factor deserving further experimental investigations to reveal potential novel targets for the prevention of metabolic diseases.

## Introduction

Obesity and diabetes are closely linked medical conditions, which, to a great extent, account for long-term morbidity and mortality worldwide [1], [2]. According to recent epidemiological evidence, both obesity and diabetes are related to chronic short sleep duration [3]. In healthy people even short-term sleep restriction or experimentally induced disturbed sleep have been shown to impair glucose tolerance [4], [5] indicating that sleep might be pivotal for metabolic homeostasis. However, very little is known about metabolic alterations in defined sleep disorders.

Tentative evidence suggests that the obstructive sleep apnea syndrome (OSAS) might be a risk factor for diabetes regardless of concomitant obesity [6]. However, in OSAS it cannot be determined whether impaired glucose tolerance is due to repeated nocturnal oxygen desaturation or due to disturbed sleep per se. In narcolepsy, increased incidence of diabetes is a long-standing finding [7], but here obesity as a confounding variable has not been ruled out [8]. Obesity has also been reported to be associated to the restless legs syndrome [9] and a relationship between RLS, diabetes [10] and impaired glucose tolerance [11] has been reported. In contrast, no studies are available on body weight or diabetes in primary insomnia. Hence, it would be of particular importance to gather more information on metabolic alterations in different sleep disorders.

To this end, we compared aspects of carbohydrate metabolism between healthy controls and patients suffering from OSAS, primary insomnia, or the restless legs syndrome (RLS). Insomnia and RLS are both highly prevalent (>5% in the general population [12], [13]) yet clearly distinct conditions of chronically disturbed sleep. Whereas primary insomnia is characterized by prominent subjective difficulties in initiating and maintaining sleep with only minor changes in objectively recorded sleep parameters [14], RLS is characterized by an impairment of sleep onset due to unpleasant feelings and an urge to move the limbs. In addition, periodic limb movements in sleep (PLMS) are typical of RLS and compromise sleep continuity to a similar extent as repeated apneic events in OSAS [15].

## Methods

### Participants

The study protocol was approved by the ethics committee of the Bavarian Medical Council, Munich, Germany. All subjects provided written informed consent prior to entering the study. Of 97 subjects investigated, 25 suffered from OSAS, 21 from primary insomnia, 18 from RLS and 33 were healthy controls. Subjects were recruited through advertisements in local newspapers. All subjects had normal findings on medical examination; they did not suffer from any psychiatric disorder and had a regular sleep-wake cycle. Subjects showing a shift of more than 2 hours during 8 days of wrist actigrapy were excluded. Pregnant women, shift workers, and persons who had travelled across multiple time zones within 3 months prior to the study were excluded. Similarly, subjects exhibiting other sleep disorders or known metabolic disorders including diabetes were excluded. All subjects showed normal results in numerous blood tests including a complete haemogram, prothrombin clotting time, activated partial thromboplastin time, fibrinogen, aspartate aminotransferase (AST/GOT) and alanine aminotransferase (ALT/GPT), LDH, lipase, cholesterol, HDL-cholesterol, LDL-cholesterol, albumin, transferrin, ferritin, iron, CRP, TSH, T3, T4, and cortisol levels, potassium, sodium, calcium, chloride, magnesium, phosphate, and urine drug monitoring. All subjects showed normal EEG findings during waking.

RLS patients met the diagnostic criteria defined by the International Restless Legs Syndrome Study Group [16] and did not suffer from any severe somatic condition such as polyneuropathy or secondary RLS. The severity of RLS was assessed using the International Restless Legs Syndrome Study Group Rating Scale (IRLS) [17]. In patients suffering from primary insomnia as well as in patients suffering from OSAS, diagnosis was based on the International Classification of Sleep Disorders [18]. OSAS patients with an apnea-hypopnea-index (AHI) above 15 (moderate sleep apnea) were included. Controls did not suffer from any sleep disturbances, and sleep related breathing disorder (AHI >5) was excluded by an ambulatory sleep apnea screening (Weinmann Somnocheck, Hamburg, Germany).

### Procedure and Measurements

Subjects underwent a detailed check-up including a physical examination, anthropometric measurements, a survey of sleep history, waking EEG recordings, and a detailed medical and psychiatric interview. In addition, sleep quality was evaluated by means of the Pittsburgh Sleep Quality Index (PSQI) [19] and daytime sleepiness using the Epworth Sleepiness Scale (ESS) [20]. To verify regular sleep-wake patterns participants were asked to wear a wrist activity monitor (Cambridge Neurotechnology, Cambridge, UK; Actiwatch Activity Analysis, Version 5.06) for 8 days prior to the study. In all patients standard nocturnal polysomnography (PSG) was conducted for two nights. Polysomnographic (PSG) recordings were performed from 23:00 to 06:00 h including monitoring of the EEG (C4-A1 and C3-A2), electrooculogram, submental electromyogram (EMG), the right and left anterior tibialis surface EMG, electrocardiogram (EKG), thoracic and abdominal movements, nasal airflow, finger oximetry, and video monitoring. Sleep stages were scored according to Rechtschaffen and Kales [21]. Sleep stages 3 and 4 were summed up to slow-wave sleep (SWS). Arousals [22], PLMS [23], and apneas/hypopneas were scored and the number of both PLMS and apneas/hypopneas per hour of total sleep time (PLMS-index and AHI, respectively) calculated. Additionally, we calculated the number of both PLMS and apneas/hypopneas associated with arousals (PLMS-arousal-index and apnea-arousal-index, respectively). Sleep stages and associated parameters were scored by two experienced scorers in each individual.

After the first night in the sleep laboratory subjects underwent a 4-hour oral glucose tolerance test (OGTT). All OGTTs were performed at 08:00 a.m. after an overnight fast. Fasting samples to assess glucose, insulin, and HbA1c were taken at baseline. After an oral standard load of 75 g glucose, blood samples were taken at 30, 60, 120, 180 and 240 min. Glucose was immediately measured using the glucose oxidase method (Synchrone DXC 800 1+2, Beckmann Coulter, USA) with an inter-assay coefficient of variation (CV) of 1.0–2.2% (DXC1) and 1.4–2.2% (DXC2). Insulin samples were measured by using an ELISA (BioSource, Germany) with an inter-assay CV of 4.2%. HbA1c measurement was based on the assessment of total Hb using the colorimetric method, the A1c concentration was determined by means of the turbidimetric immuno-inhibition method (Synchrone DXC 800 1, Beckmann Coulter, USA) with a inter-assay CV of 2.9–3.2%. Standardized to the NGSP reference range was set at 4.3–5.8%.

According to the diagnostic criteria of the American Diabetes Association [24] we defined normal glucose tolerance (NGT) as 2-hour plasma glucose (2h-PG) concentrations (2 h after an oral glucose challenge) <140 mg/dl; impaired glucose tolerance was defined as 2h-PG values ≥140 and diabetes as 2h-PG ≥200 mg/dl. The total areas under the curve for glucose (AUCg) and insulin (AUCi) were calculated using the linear trapezoidal rule [25]. The combination of elevated HbA1c values (>5.5%) and impaired fasting glucose values (FPG >100 mg/dl) was assessed as an additional risk factor for the development of type 2 diabetes, since several studies have shown that the combination of these parameters is a stronger predictor for the risk of developing type 2 diabetes than increased fasting glucose alone [26]–[29]. Insulin resistance was assessed using the homeostasis model assessment (HOMA-IR) originally described by Mathew et al. [30] HOMA-IR was calculated using the following equation: HOMA-IR (mg/dl × µU/ml) = FPG mg/dl × FPI µU/ml/405. To encompass both hepatic and peripheral insulin sensitivity we calculated a composite measure of whole body insulin sensitivity (ISIcomposite) recommended by Matsuda et al. [31] (ISIcomposite was calculated using the following formula: ISIcomposite = 10 000/√ ((FPG × FPI) × (mean OGTT glucose × mean OGTT insulin)).

### Statistical Analysis

Statistical analysis was performed using SPSS for Windows 16.0 (SPSS Inc., Chicago, Illinois). Using Gabriel- or Games-Howell-corrected analysis of variance (ANOVA) tests we compared the mean values of the basic characteristics age, BMI, PSQI and ESS among the four groups. Because of their skewed distribution, HbA1c, FPG, 2h-PG, fasting plasma insulin (FPI), the area under the curve for glucose (AUCg) and the area under the curve for insulin (AUCi) were z-transformed. On metabolic parameters analysis of covariance (ANCOVA) was conducted with the BMI as covariate.

A chi-square test was performed to compare the incidence of IGT and/or combined elevated HbA1c and FPG values between groups. After constructing 2×2 contingency tables odds ratios (OR) were calculated. First-order partial correlation and bivariate correlation, respectively, were done to determine the association between continuous variables. *P*<0.05 was considered as statistically significant.

## Results

Table 1 shows the baseline characteristics for all subjects. Groups did not differ with respect to age. As expected, OSAS patients had a strongly increased BMI, whereas the BMI of RLS and insomnia patients did not differ from controls. RLS patients suffered from a severe restless legs syndrome (IRLS, 22.9±5.4). OSAS patients were all males, yielding a statistically significant gender difference between groups. RLS and primary insomnia patients, however, did not show gender distributions different from controls.

**Table 1:** Baseline Characteristics of Study Participants.

	OSAS	RLS	INS	CON	P-value
**Females/Males**	0/25 ‡ ∇	11/7	12/9	17/16	<0.001
**Age (years)**	52.3 (10.8)	52.2 (13.0)	49.1 (9.7)	46.8 (7.7)	>0.05
**BMI (kg/m^2^)**	32.9 (5.4) ‡	25.4 (3.7)	25.0 (4.8)	24.7 (3.5)	<0.001
**PSQI**	6.7 (2.8) ‡ ∇	9.3 (4.5) ‡ ^+^	13.1 (3.7) ‡	3.0 (2.0)	<0.001
**ESS**	11.2 (5.6) ‡ □	7.9 (4.3)	6.4 (4.0)	6.8 (2.8)	<0.001

Data are mean (SD). Statistical comparison was done using Gabriel- or Games-Howell-corrected oneway ANOVA.

OSAS, Obstructive sleep apnea syndrome; RLS, Restless legs syndrome; INS, Insomnia; CON, controls; BMI, Body mass index; PSQI, Pittsburgh Sleep Quality Index; ESS, Epworth Sleepiness Scale.

* p<0.05, † p<0.01, ‡ p<0.001, vs controls.

^+^ p<0.05, □ p<0.01, ∇ p<0.001, between groups.

OSAS: χ^2^ (1) = 18.21, p<0.001; RLS: χ^2^ (1) = 0.433, p>0.05; INS: χ^2^ (1) = 0.163, p>0.05, vs controls.

OGTT data indicated that no control and no primary insomnia patient, but four OSAS patients and one RLS patient suffered from diabetes. As shown in Table 2, 12% of the controls showed 2h-PG values indicating an impaired glucose tolerance. This rate was significantly increased in OSAS (40%, OR 4.9) and RLS (39%, OR 4.7) patients, but not in primary insomnia (18%, OR 1.6) patients. The rate of impaired glucose tolerance within groups was not gender dependent (data not shown). As shown in Table 3, mean HbA1c values were in the normal range (below 5.8%), but differed significantly between groups after adjustment for the BMI. A Sidak-corrected post-hoc test based on estimated marginal means revealed that OSAS as well as RLS patients had significantly higher HbA1c values than healthy controls. In both OSAS and RLS patients the rate of patients displaying elevated HbA1c and FPG was significantly increased. Accordingly, 56% of the OSAS (OR 20.0) and 35% of the RLS (OR 8.5) patients belonged to the high risk group for developing type 2 diabetes. In contrast, only 5% of the insomniacs (OR 0.82) and 6% of the controls showed elevated HbA1c plus FPG levels (see Table 4).

**Table 2:** Frequency of Patients with Normal Glucose Tolerance or Impaired Glucose Tolerance.

	Total	Normal glucose tolerance	Impaired glucose tolerance	Odds ratio
	N	N (%)	N (%)	
**OSAS**	25	15 (60)	10 (40)	4.9
**RLS**	18	11 (61)	7 (39)	4.7
**INS**	17	14 (82)	3 (18)	1.6
**CON**	32	28 (88)	4 (12)	

Normal Glucose Tolerance: 2h-PG≤140 mg/dl; Impaired Glucose Tolerance: 2h-PG≥140 mg/dl.

OSAS, Obstructive sleep apnea syndrome; RLS, Restless legs syndrome; INS, Insomnia; CON, controls.

χ^2^ (3) = 7.95, p<0.05.

**Table 3:** Metabolic Parameters.

	OSAS	RLS	INS	CON	BMI-adjusted P-value
**HbA1c (%)**	5.6 (0.4) †	5.5 (0.3) †	5.3 (0.3)	5.2 (0.3)	<0.01
**FPG (mg/dl)**	110.9 (15.6)	100.4 (10.1)	98.8 (8.9)	96.0 (8.0)	>0.05
**FPI (µl/ml)**	16.2 (10.5)	8.7 (3.2)	10.3 (5.4)	8.9 (3.3)	>0.05
**2h-PG (mg/dl)**	139.0 (55.7)	121.1 (40.8)	120.2 (17.8)	109.5 (23.k5)	>0.05
**2h-PI (µl/ml)**	82.5 (67.3)	60.0 (67.8)	50.6 (21.3)	36.8 (29.2)	>0.05
**AUCg (mg/dl)**	31882 (7592)	28063 (5748)	28369 (3500)	25859 (4014)	>0.05
**AUCi (µl/ml)**	15024 (9396)	9873 (6921)	8437 (3588)	8307 (5258)	>0.05
**HOMA1-IR**	4.7 (3.8)	2.2 (1.0)	2.5 (1.3)	2.1 (0.9)	>0.05
**ISIcomposite**	3.1 (1.6)	6.3 (5.4)	5.1 (1.5)	5.5 (1.9)	>0.05

Data are mean (SD). Statistical comparison was done using ANCOVA.

OSAS, Obstructive sleep apnea syndrome; RLS, Restless legs syndrome; INS, Insomnia; CON, controls; FPG = Fasting plasma glucose; FPI = Fasting plasma insulin; 2h-PG = 2h-Postload glucose; 2h-PI = 2h-Postload insulin; AUCg = Area under the curve for glucose; HOMA1-IR = Homeostasis model assessment-1 of insulin resistance; ISIcomposite = Insulin sensitivity index composite.

* p<0.05, † p<0.01, ‡ p<0.001.

**Table 4:** Frequency of Patients with Normal or Elevated HbA1c (≥5.5%) & FPG (≥100mg/dl).

	Total	Normal HbA1c&FPG	Elevated HbA1c&FPG	Odds ratio
	N	N (%)	N (%)	
**OSAS**	25	11 (44)	14(56)	20.0
**RLS**	17	11 (65)	6 (35)	8.5
**INS**	19	18 (95)	1 (5)	0.8
**CON**	33	31 (94)	2 (6)	

OSAS, Obstructive sleep apnea syndrome; RLS, Restless legs syndrome; INS, Insomnia; CON, controls; FPG = Fasting plasma glucose.

χ^2^ (3) = 25·31, p<0·001.

Daytime sleepiness (ESS) was highest in OSAS patients and did not differ in the other patient groups from controls. In contrast, night sleep was subjectively impaired in all three patient groups compared to controls as assessed by the PSQI, and insomniacs rated their sleep significantly worse than did OSAS and RLS patients (Table 1). Patient groups did not differ significantly in the total amount and efficiency of polysomnographically defined sleep. Similarly, the amount of wakefulness during the night was comparable between groups. OSAS patients showed less SWS and REM sleep, but more stage 1 sleep than both other patients groups. Due to the selection criteria of the present study, only OSAS patients showed pathological apnea-hypopnea and oxygen desaturation indices. Mean PLMS arousal indices were in the pathological range in OSAS and RLS patients, but not in insomnia patients (Table 5).

**Table 5:** Sleep Parameters.

	OSAS	RLS	INS	P-value
**TIB (min)**	437.5 (33.1)	449.2 (32.8)	435.7 (25.8)	>0.05
**TST (min)**	339.0 (58.7)	345.9 (69.5)	337.3 (55.6)	>0.05
**SEI (%)**	77.5 (11.8)	77.1 (14.6)	77.2 (10.8)	>0.05
**WASO (min)**	75.7 (42.8)	75.3 50.1)	61.0 (35.6)	>0.05
**REM (min)**	41.4 (18.5) ‡	73.0 (24.1)	70.8 (27.2)	<0.001
**S1 (min)**	92.2 (40.2) ‡	41.8 (20.0)	31.3 (14.5)	<0.001
**S2 (min)**	198.0 (47.9)	204.2 (47.9)	214.9 (39.5)	>0.05
**SWS (min)**	7.4 (9.5) †	26.9 (29.1)	20.4 (17.5)	<0.01
**SO (min)**	21.2 (20.9)	22.3 (33.2)	22.4 (19.6)	>0.05
**SLREM (min)**	137.7 (68.4) *	97.5 (72.8)	86.4 (46.9)	<0.05
**SLSWS (min)**	122.8 (104.9) *	67.9 (92.6)	37.3 (26.0)	<0.05
**AHI**	55.7 (27.1) ‡	3.3 (3.7)	1.6 (1.6)	<0.001
**ODI**	43.6 (29.7) ‡	1.9 (3.0)	1.4 (1.6)	<0.001
**PLMS-Index**	19.2 (20.4)	32.5 (33.1)	5.2 (7.2) †	<0.01
**PLMS-arousal-index**	12.8 (14.0)	14.8 (15.3)	2.8 (4.7) †	<0.01
**Apnea-arousal-index**	33.8 (25.6) ‡	0.8 (1.8)	0.3 (0.5)	<0.001

Data are mean (SD). Mean comparison was done using Gabriel- or Games-Howell-corrected oneway ANOVA.

OSAS, Obstructive sleep apnea syndrome; RLS, Restless legs syndrome; INS, Insomnia; CON, controls; TIB, Time in bed; TST, Total sleep time; SEI, Sleep efficiency; WASO, Wake time after sleep onset; REM, Rapid eye movement sleep; S1, Stage 1 sleep; S2, Stage 2 sleep, SWS, Slow wave sleep; SO, Sleep onset latency; SLREM, REM sleep latency; SLSWS, SWS sleep latency; AHI, Apnea-hypopnea-index; ODI, Oxygen-desaturation-index; PLMS-Index, Periodic leg movements per hour of sleep; PLMS-arousal-index, PLMS associated with arousals per hour of sleep; Apnea-arousal-index, Apneas and/or hypopneas associated with arousals per hour.

* p<0·05, † p<0·01, ‡ p<0·001.

**Figure 1:**
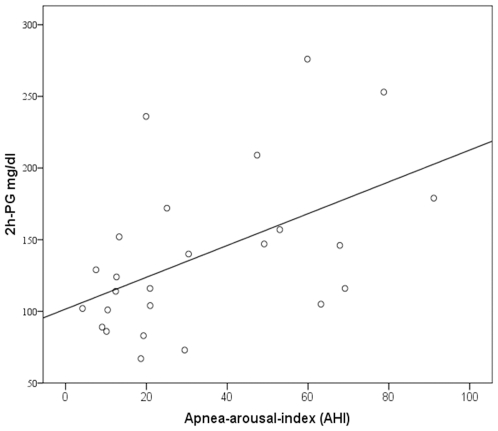
Association between 2 hour-plasma glucose and the apnea-arousal-index in obstructive sleep apnea patients. Partial correlation coefficient (corrected for the BMI); r = 0.56, *p*<0.05.

Tasali et al. [5] have shown that SWS suppression induced by acoustic stimulation triggering repetitive microarousals leads to a decrease in insulin sensitivity. Because OSAS and RLS are characterized by repeated arousals due to apneas and PLMS, we assessed whether they were related to those metabolic parameters differing between patient groups. 2h-PG values correlated positively with the apnea-arousal-index (r = 0.56, *p*<0.05) and the ODI (r = 0.59, *p*<0.05) in OSAS, and with the PLMS-arousal-index (r = 0.56, *p*<0.05) in RLS (Figures 1–3). HbA1c correlated positively with the apnea-arousal-index (r = 0.50, *p*<0.05) in OSAS, which was neither the case for the ODI (r = 0.46, *p*>0.05), nor for the PLMS-arousal-index (r = 0.21, *p*>0.05) in RLS.

**Figure 2 pone-0009444-g002:**
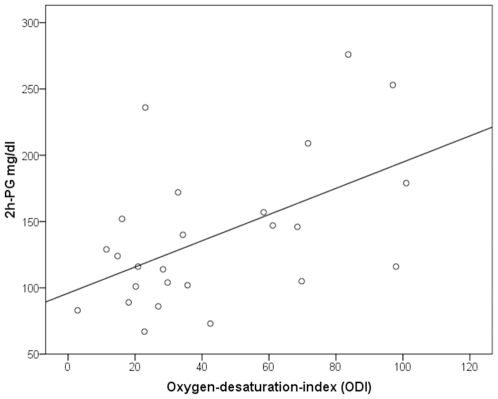
Association between 2 hour-plasma glucose and the oxygen-desaturation-index in obstructive sleep apnea patients. Partial correlation coefficient (corrected for the BMI); r = 0.59, *p*<0.05.

**Figure 3:**
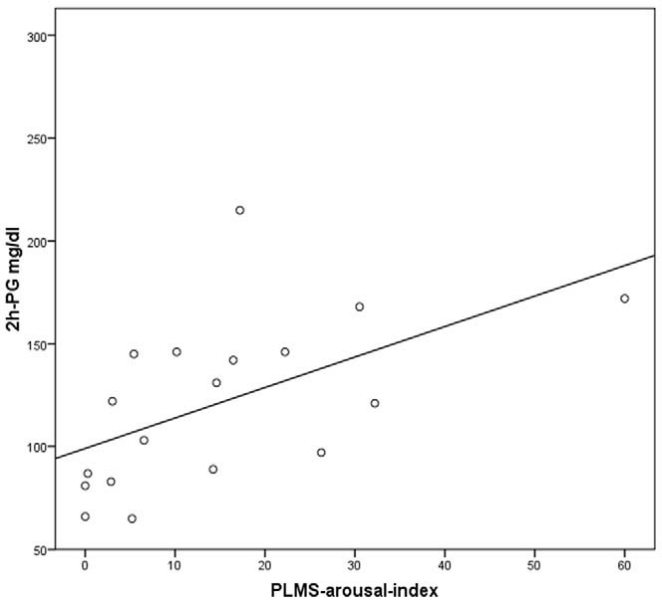
Association between the 2 hour-plasma glucose and the PLMS-arousal-index in restless legs patients. Pearson's correlation coefficient; r = 0.56, *p*<0.05.

## Discussion

The present study for the first time compared glucose metabolism in various sleep disorders. The major finding is an increased rate of impaired glucose tolerance in patients with OSAS and RLS, but not in patients with primary insomnia, as compared to normal controls.

Both RLS and OSAS patients were almost five times more likely to suffer from impaired glucose tolerance than healthy controls. In addition, mean HbA1c values were increased in both diagnostic groups compared to controls, and the rate of values above the reference level in both HbA1c and fasting plasma glucose levels indicated an increased diabetes risk in patients with OSAS (20-fold) and RLS (9-fold), respectively. The control group was not matched to the OSAS group with respect to gender. Indeed OSAS patients were exclusively males. Hence, the present results should, as far as OSAS is concerned, be replicated in a gender-mixed patient group compared to gender-matched controls.

Impaired glucose tolerance and increased rates of diabetes have been frequently shown in patients with OSAS [32] and might be related, to a great extent, to obesity, which was also documented in the present study by an increased BMI in this patient group. However, some studies suggest that OSAS is related to type 2 diabetes independently of obesity [33]. The idea has been put forward that nocturnal breathing disorders comprise glucose metabolism either due to repeated oxygen desaturation or due to disturbed sleep per se.

The present study suggests that indeed disturbed sleep itself detoriates glucose metabolism, as non-obese RLS patients devoid of any nocturnal breathing problem showed impaired glucose tolerance to the same extent as OSAS patients. The positive correlation between quantitative measures of sleep interruptions (apnea-arousal-index in OSAS and PLMS-arousal-index in RLS) and 2h-plasma glucose levels in the OGTT indicate repeated arousals as a possible common mechanism of impaired glucose tolerance in both sleep disorders. This idea is in line with the report of Tasali [5], who induced impaired glucose tolerance in healthy volunteers by disturbing sleep by means of acoustical stimulation for three consecutive nights. This procedure did not alter the total amount of sleep, suggesting a specific effect of repeated arousals. Our results support this idea, because patients with primary insomnia and normal glucose tolerance had similar total sleep times compared to OSAS and RLS patients, but lacked the repeated arousals induced by apneas or PLMS.

Repeated arousals could affect glucose metabolism by altering sleep structure, in particular by preventing SWS. This is suggested by a correlation between SWS and insulin sensitivity in the study of Tasali [5]. Repeated arousals, however, could also have a direct effect on glucose metabolism by the resulting activation of the sympathetic nervous system. Increases in heart rate and blood pressure are typical of both obstructive apneas and periodic leg movements, and sympathetic nervous system activation has been shown in both OSAS and RLS patients [34], [35].

Because we restricted polysomnographic recordings to the patient groups, the present study does not help to directly answer the question whether sleep duration or other quantitative aspects of night sleep, independently of arousing stimuli during sleep, affect glucose metabolism. Indirectly, similar sleep durations of 5.5 hours in all our patient groups, including primary insomnia patients who showed no impairment in glucose tolerance, suggests that only more prominent sleep reduction might have a direct negative effect on carbohydrate metabolism. One example would be sleep curtailment to four hours of sleep for some days [4], which impaired glucose metabolism even in healthy people.

RLS is frequently found in patients with diabetes, which so far has mainly been explained by the fact that diabetes-induced polyneuropathy predisposes to RLS [10]. In line with this argument, Bosco et al. [11] found an increased incidence of small fiber neuropathy (SFN) in RLS patients with impaired glucose tolerance. Because we did not perform skin biopsies, we can not judge the role of SNF in our sample. But, the present study for the first time suggests that not only diabetes might predispose to RLS through SNF or other mechanisms, but that in turn, RLS might be a causative factor in the development of diabetes by compromising sleep continuity due to repeated arousals. Moreover, the present results further strengthen the idea that disturbed sleep in general and chronic sleep disorders in particular might significantly contribute to the steady increase in diabetes prevalence worldwide. This calls for further extended investigations, because sleep disorders are highly prevalent and might represent an important preventive target to avoid metabolic disorders.
